# Entropy-based analysis of Rift Valley fever transmission dynamics using delay differential equations

**DOI:** 10.1371/journal.pone.0341046

**Published:** 2026-02-05

**Authors:** Ali Raza, Mansoor Alsulami, Marek Lampart, Umar Shafique, Eman Ghareeb Rezk

**Affiliations:** 1 IT4Innovations, VSB-Technical University of Ostrava, Ostrava, Czech Republic; 2 Jadara University Research Center, Jadara University, Jordan; 3 Department of Mathematics, Faculty of Science, King Abdulaziz University, Jeddah, Saudi Arabia; 4 Department of Mathematics, National College of Business Administration and Economics, Lahore, Pakistan,; 5 Mathematical science department, College of science, Princess Nourah bint Abdlrahman University, Riyadh, Saudi Arabia; Central Laboratory for Evaluation of Veterinary Biologics, Agricultural Research Center, EGYPT

## Abstract

Rift Valley Fever (RVF) is a vector-borne, transmissible zoonotic disease in animals and humans caused by RVF virus and transmitted primarily by mosquitoes and biting flies. According to the World Health Organization (WHO), RVF outbreaks have great public health and economic impact, with up to 90% mortality in lambs and approximately 10% in adult sheep. Besides small ruminants, RVF affects cattle, camels, and goats and induces abortion storms and neonatal mortality at high rates, whereas humans develop severe febrile illness, hemorrhagic fever, or encephalitis in extreme cases. In this study, a deterministic epidemic model is developed and studied to investigate the RVF transmission dynamics under inclusion of delay differential equations and an entropy-based global stability approach to capture the time-dependent effect of disease progression. The model separates the ruminant population into susceptible, vaccinated, infected, and recovered, and the mosquito population into susceptible and infected. Positivity, and boundedness are demonstrated as basic dynamical properties. Two equilibria are found and compared: Rift Valley Fever–Free Equilibrium (RVFFE) and Rift Valley Fever Endemic Equilibrium (RVFEE). The basic reproduction number ($R_{0}$) is derived and local and entropy-based global stability are examined by the Routh–Hurwitz criteria and LaSalle’s invariance principle. Sensitivity analysis reveals the contribution of critical parameters on disease transmission and persistence. Numerical simulations performed using MATLAB’s built-in DDE23 solver assist in revealing the effect of delay on infection dynamics and susceptibility with time. The implications provide tremendous insight into the role of temporal considerations and parameter uncertainty in the transmission dynamics of RVF and enable better understanding as well as potential control strategies for this emerging zoonotic disease.

## 1 Introduction

Rift Valley fever (RVF) is a highly infectious viral disease that spreads rapidly among animal populations and can cause catastrophic outbreaks. RVF is a mosquito-borne zoonosis, affecting major domestic ruminant species like sheep, cattle, goats, and camels. Humans usually develop symptoms through infected mosquito bites or by contact with tissues from an infected animal. The dynamics are dependent on the interaction between susceptible ruminants and infected female mosquitoes that act as vectors of the virus. Infected ruminants develop high viremia and efficiently transmit the virus to mosquitoes while they feed on the animal’s blood. In turn, infected mosquitoes remain infectious for their lifetime, providing continuity to the host–vector transmission cycle. Indeed, RVF outbreaks often strike with surprising suddenness throughout the endemic areas when favorable environmental and climatic conditions take place and mosquitoes spread. Vaccination and recovery remove the hosts from the pool of infective individuals, but vaccine-induced immunity may wane with time, returning individuals to the susceptibility pool, while infected ruminants either recover or die from disease-related complications. Time delays naturally arise from incubation periods, immune responses, and delayed intervention effects and may significantly affect the timing of outbreaks and disease persistence. It is thus important to incorporate such delays when accurately describing RVF dynamics and assessing effective control strategies. Several research studies have developed mathematical models to understand and control its spread dynamics. For instance, [1] modeled RVF as a rapidly spreading viral disease and proposed a vaccination-based mathematical model for managing the disease, while [2] presented another modeling approach for virus control in the population. Investigation in [3] was directed at the role of mosquito bites in the transmission of RVF, emphasizing the resulting economic losses in affected regions and also the usefulness of mathematical modeling in disease control. Further, [4] described efforts at raising awareness and organizing prevention measures such as vaccination and treatment during major outbreaks.

In [5], a predictive modeling approach was proposed for the estimation of future RVF transmission within the population. The contribution of climatic and environmental factors to the transmission of RVF was highlighted in [6] through the proof that epidemic prevention can be achieved using weather forecasting. In [7], the author studied a computational analysis of stochastic delay dynamics in maize streak virus. Similarly, [8] presented a dynamical model of viral dynamics in animal hosts.

Studies in [9] explored the heightened susceptibility of drug-addicted animals to RVF via weakened immune systems, with the inference that decreased drug exposure can limit disease spread. Prevention strategies were also discussed in [10], which provided effective control measures. The impact of global warming on RVF epidemics was investigated in [11], showing that elevated temperatures amplify infection severity. A computational analysis aimed at African outbreaks was introduced in [12], and it suggested several strategies for mitigating RVF transmission in livestock populations.

Modeling approaches in [13,14] prioritized the contagiousness of RVF and its effects on animal blood cells, applying high-level technologies for outbreak prediction and control. The work in [15] prioritized how RVF infects the circulatory and immune systems, with treatment-based control. Camel infections and camel immune reactions were the research priority in [16], while [17] accounted for parameter uncertainty in RVF models and demonstrated sensitive parameter targeting to suppress infection.

Further RVF transmission mathematical models were shown in [18,19], both of which also showed predictive findings and strategies for epidemic control. Finally, [20] addressed diagnosis and prevention of RVF outbreaks in animals, while [21] discussed impulsive reaction–diffusion delayed models in biological systems with an emphasis on the importance of time delays to model disease dynamics. Mathematical modeling has been widely exploited in conducting analysis of infectious disease transmission dynamics and assessing the impact of control strategies such as vaccination, treatment, and environmental interventions [22]. Deterministic, fractional-order, and optimal control frameworks have been recently used to explore some viral and parasitic diseases under realistic epidemiological settings; the approaches are proven to be viable in informing public-health decision making [23–27]. Most of the existing models on RVF tend to ignore time delays associated with incubation, immune response, and delayed interventions in the dynamics of transmission and persistence of outbreaks. In this paper, we formulate a delayed host-vector model and investigate the long-term dynamics and implications of interventions for Rift Valley Fever using an entropy-based global stability framework.

In biomathematics, delay is also very important in describing disease behavior and transmission dynamics. A more realistic form of deterministic modeling is thus realized by including time delays. From this research, besides medical intervention, the best way to control Rift Valley Fever infection is by using preventive and protective measures. Motivated by this, the present work introduces a delay-based modeling framework to capture the temporal effects of infection and control strategies. Moreover, driven by such biological and epidemiological properties, we propose a mathematical model employing a delay differential equation which reflects the host-vector transmission and the effect of Rift Valley Fever.

The remainder of this paper is organized as follows. Section 2 presents the formulation of the delayed model for Rift Valley Fever and analyzes its basic dynamical properties such as positivity and boundedness. Section 3 derives the model equilibria and the basic reproduction number. Section 4 investigates the local and entropy-based global stability of an equilibrium. Section 5 provides a sensitivity analysis of key parameters affecting disease transmission. Section 6 presents the numerical simulations based on the MATLAB DDE23 solver to illustrate the dynamic behavior of the model. Finally, Section 7 concludes the paper and outlines future research directions.

## 2 Structure of model

**Fig 1:**
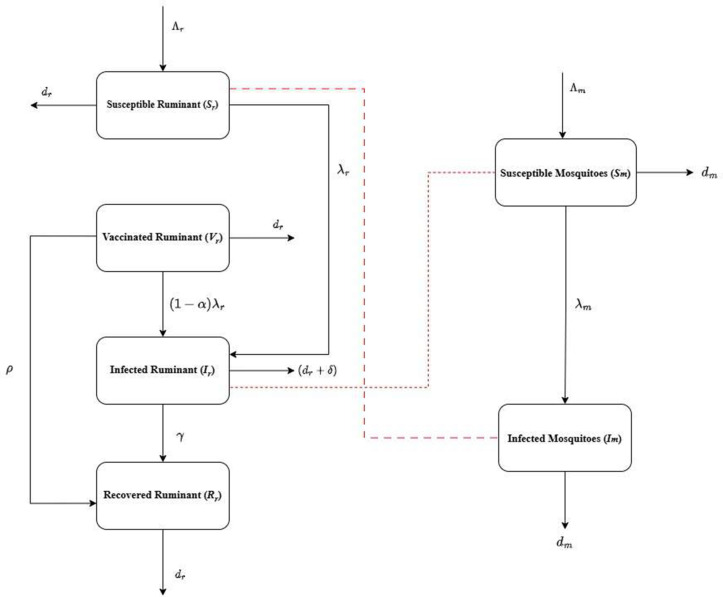
Flow chart of Rift Valley fever dynamics.

In this section, we discussed the formulation of the delayed Rift Valley fever deterministic epidemic model. In this model, there are two different populations, which are female mosquitoes and ruminants. The first $N_{r}$, which is the total population of ruminants and the total population of female mosquitoes is categorized as $N_{m}$. The ruminant population is divided into different compartments, which are susceptible $"S_{r}"$, vaccinated “$V_{r}$”, infected $"I_{r}"$, and recovered $"R_{r}"$, while the total population of female mosquitoes is divided into susceptible “$S_{m}$” and infected class $"I_{m}"$ (See Fig 1).

All model parameters are summarized in Table 1, along with their biological interpretation, value, and unit. Unless otherwise stated, all parameter values have been adopted from the literature [21] and were consistently used throughout the numerical simulations.

**Table 1:** Model parameters, biological descriptions, values, and units.

Parameter	Description	Value	Units
$\beta_{r}$	Transmission rate from infected mosquitoes to ruminants	0.14 (RVFFE); 2.14 (RVFEE)	${\text{day}}^{-\text{1}}$
b	Mosquito biting rate	0.0701 (RVFFE); 0.701 (RVFEE)	${\text{day}}^{-\text{1}}$
$\beta_{m}$	Transmission rate from infected ruminants to mosquitoes	0.35	${\text{day}}^{-\text{1}}$
$d_{m}$	Natural death rate of mosquitoes	0.5	${\text{day}}^{-\text{1}}$
$d_{r}$	Natural death rate of ruminants	0.5	${\text{day}}^{-\text{1}}$
$\Lambda_{m}$	Recruitment rate of mosquitoes	0.5	${\text{day}}^{-\text{1}}$
$\Lambda_{r}$	Recruitment rate of ruminants	0.5	${\text{day}}^{-\text{1}}$
α	Vaccine efficacy rate	0.05	${\text{day}}^{-\text{1}}$
ρ	Waning rate of vaccine-induced immunity	0.001	${\text{day}}^{-\text{1}}$
γ	Recovery rate of infected ruminants	0.875	${\text{day}}^{-\text{1}}$
v	Vaccination rate of susceptible ruminants	0.481	${\text{day}}^{-\text{1}}$
δ	Disease-induced death rate of infected ruminants	0.5	${\text{day}}^{-\text{1}}$

The Rift Valley Fever transmission model presented below has been derived on the following assumptions:

- The populations of ruminant hosts and of female mosquitoes are homogeneously mixed with no spatial or age structure.
- The disease can be transmitted through bites by female disease-carrying mosquitoes and through infection of the mosquito by feeding on diseased ruminants.
- The rates of recruitment and natural death of ruminants and mosquitoes remain constant.
- Vaccination is only used on susceptible ruminants and offers partial protection, with immunity gradually decreasing over time.
- The infectious ruminants can either recover with a fleeting immunity or die from disease causing mortality.
- The infected mosquitoes will always be infectious and will not recover.
- The time delay represents the incubation or progress phases of the infectious disease, in which there may be a natural death rate.
- Environmental, seasonal, and stochastic factors are indirectly accounted for in the current formulation.

Define current forces of infection:


$$\lambda_{r}(t)=b\;\beta_{r}\;I_{m}(t),\lambda_{m}(t)=b\;\beta_{m}\;I_{r}(t).$$

Use host and vector delays $\tau_{r},\tau_{m}\geq \text{0}$ (set either to 0 if not used). Then:


$$\begin{array}{cc} \frac{dS_{r}}{dt} & \;=\Lambda_{r}-\lambda_{r}(t)\;S_{r}(t)-(v+d_{r})S_{r}(t),\\ \frac{dV_{r}}{dt} & \;=v\;S_{r}(t)-(\text{1}-\alpha )\lambda_{r}(t)\;V_{r}(t)-(\rho +d_{r})V_{r}(t),\\ \frac{dI_{r}}{dt} & \;=\lambda_{r}(t-\tau )[S_{r}(t-\tau )+(\text{1}-\alpha )V_{r}(t-\tau )]e^{-d_{r}\tau }-(d_{r}+\gamma +\delta )I_{r}(t),\\ \frac{dR_{r}}{dt} & \;=\rho \;V_{r}(t)+\gamma \;I_{r}(t)-d_{r}R_{r}(t),\\ \frac{dS_{m}}{dt} & \;=\Lambda_{m}-\lambda_{m}(t)\;S_{m}(t)-d_{m}S_{m}(t),\\ \frac{dI_{m}}{dt} & \;=\lambda_{m}(t-\tau )\;S_{m}(t-\tau )\;e^{-d_{m}\tau }-d_{m}I_{m}(t). \end{array}$$ (1)

Here, $\lambda_{r}$ is the per-capita infection pressure on ruminants due to infectious mosquitoes, and $\lambda_{m}$ is the per-capita infection pressure on mosquitoes due to infectious ruminants.

Survival-adjusted constants for delayed inflows.


$$K_{\text{1}}=b\beta_{r}\;e^{-d_{r}\tau },K_{\text{3}}=(\text{1}-\alpha )\;b\beta_{r}\;e^{-d_{r}\tau },K_{\text{6}}=b\beta_{m}\;e^{-d_{m}\tau },$$

$$\;K_{\text{2}}=v+d_{r},K_{\text{4}}=\rho +d_{r},K_{\text{5}}=d_{r}+\gamma +\delta .$$

In incorporating the time delay, we make a distinction between the time of infection and the time when the individuals enter the infected class. The forces of infections given in (1) reflect the incidence at the current time, whereas the new infectives occur after a certain time period. τ, indicating incubation or duration of progression. During the waiting period, natural deaths occur; hence, only the surviving proportion adds to the infectious segment. This takes place through exponential survival multipliers of the form $e^{-d_{r}\tau }$, where d: this represents the natural death rate. Accordingly, the exponential terms involving the delayed inflow rates in equations (2–7) are multiplicatively adjusted to reflect the survival rates. The depletion of $S_{r}$ and $V_{r}\;$uses the current force of infection, while the appearance of new infectious individuals uses the delayed incidence multiplied by the appropriate survival factor:


$$\frac{dS_{r}}{dt}=\Lambda_{r}-\lambda_{r}(t)S_{r}(t)-K_{\text{2}}S_{r}(t),$$ (2)

$$\frac{dV_{r}}{dt}=vS_{r}(t)-(\text{1}-\alpha ){\lambda_{r}(t)V}_{r}(t)-K_{\text{4}}V_{r}(t),$$ (3)

$$\frac{dI_{r}}{dt}=K_{\text{1}}S_{r}(t-\tau )I_{m}(t-\tau )+K_{\text{3}}V_{r}(t-\tau )I_{m}(t-\tau )-K_{\text{5}}I_{r}(t),$$ (4)

$$\frac{dR_{r}}{dt}=\rho V_{r}(t)+\gamma I_{r}(t)-d_{r}R_{r}(t),$$ (5)

$$\frac{dS_{m}}{dt}=\Lambda_{m}-\lambda_{m}(t)S_{m}(t)-d_{m}S_{m}(t),$$ (6)

$$\frac{dI_{m}}{dt}=K_{\text{6}}S_{m}(t-\tau )I_{r}(t-\tau )-d_{m}I_{m}(t).$$ (7)

Feasible invariant set and histories. Let $N_{r}=S_{r}+V_{r}+I_{r}+R_{r}$ and $N_{m}=S_{m}+I_{m}$. Assuming nonnegative, bounded history functions on [−τ,0],


$$(S_{r},V_{r},I_{r},R_{r},S_{m},I_{m})\in C([-\tau ,\text{0}],R_{\geq \text{0}}^{\text{6}}),$$

the feasible invariant region is


$$\begin{array}{c} V=\{(S_{r},V_{r},I_{r},R_{r},S_{m},I_{m})\in R_{\geq \text{0}}^{\text{6}}:N_{r}\leq \frac{\Lambda_{r}}{d_{r}},N_{m}\leq \frac{\Lambda_{m}}{d_{m}}\}. \end{array}$$

***Theorem 1****.* Consider system (2–7) with a single delay τ≥0 and nonnegative, continuous history functions on [−τ,0]:


$$(S_{r},V_{r},I_{r},R_{r},S_{m},I_{m})\in C([-\tau ,\text{0}],R_{\geq \text{0}}^{\text{6}}).$$

Then its solution remains nonnegative for all t≥0 and stays in the feasible region


$$\begin{array}{c} V\;\;=\{(S_{r},V_{r},I_{r},R_{r},S_{m},I_{m})\in {\mathbb{R}}_{\geq \text{0}}^{\text{6}}:\;N_{r}\leq \frac{\Lambda_{r}}{d_{r}},\;\;N_{m}\leq \frac{\Lambda_{m}}{d_{m}}\}, \end{array}$$

where $N_{r}=S_{r}+V_{r}+I_{r}+R_{r}$ and $N_{m}=S_{m}+I_{m}$.

***Proof:*** At any time t≥0, if a component hits zero, we evaluate its derivative on the boundary, using that all delayed arguments are nonnegative by the history assumption and positivity up to time t.

Using (2–7):

- At $S_{r}(t)=\text{0}$:$\frac{dS_{r}}{dt}{|}_{S_{r}=\text{0}}=\Lambda_{r}\geq \text{0}.$
- At $V_{r}(t)=\text{0}$:$\frac{dV_{r}}{dt}{|}_{V_{r}=\text{0}}=v\;S_{r}(t)\geq \text{0}.$
- At $I_{r}(t)=\text{0}$:$\frac{dI_{r}}{dt}{|}_{I_{r}=\text{0}}=K_{\text{1}}\;S_{r}(t-\tau )I_{m}(t-\tau )+K_{\text{3}}\;V_{r}(t-\tau )I_{m}(t-\tau )\geq \text{0}.$
- At $R_{r}(t)=\text{0}$:$\frac{dR_{r}}{dt}{|}_{R_{r}=\text{0}}=\rho \;V_{r}(t)+\gamma \;I_{r}(t)\geq \text{0}.$
- At $S_{m}(t)=\text{0}$:$\frac{dS_{m}}{dt}{|}_{S_{m}=\text{0}}=\Lambda_{m}\geq \text{0}.$
- At $I_{m}(t)=\text{0}$:$\frac{dI_{m}}{dt}{|}_{I_{m}=\text{0}}=K_{\text{6}}\;S_{m}(t-\tau )I_{r}(t-\tau )\geq \text{0}.$

Thus, no component can cross the boundary into negative values; the nonnegative cone is positively invariant. The bounds $N_{r}\leq \frac{\Lambda_{r}}{d_{r}}$ and $N_{m}\leq \frac{\Lambda_{m}}{d_{m}}$ are shown in Theorem 2

***Theorem 2*** For system (2–7) with nonnegative histories, the total host and vector populations satisfy


$$\text{0}\leq N_{r}(t)\leq \frac{\Lambda_{r}}{d_{r}}+(N_{r}(\text{0})-\frac{\Lambda_{r}}{d_{r}})e^{-d_{r}t},\text{0}\leq N_{m}(t)\leq \frac{\Lambda_{m}}{d_{m}}+(N_{m}(\text{0})-\frac{\Lambda_{m}}{d_{m}})e^{-d_{m}t},$$

hence $N_{r}(t)\leq \frac{\Lambda_{r}}{d_{r}}$ and $N_{m}(t)\leq \frac{\Lambda_{m}}{d_{m}}$ for all t≥0.

***Proof:*** Sum the host equations (2–5):


$$N_{r}^{\prime }=S_{r}^{\prime }+V_{r}^{\prime }+I_{r}^{\prime }+R_{r}^{\prime }$$

$$\begin{array}{cc} =\; & \;\Lambda_{r}-\;\;\;\;\;\underset{\underbrace{current\;incidence}}{\lambda_{r}(t)S_{r}(t)}\;\;\;\;-(v+d_{r})S_{r}(t)+vS_{r}(t)-(1-\alpha )\lambda_{r}(t)V_{r}(t)-(\rho +d_{r})V_{r}(t)\\ & \;\;\;\underset{\underbrace{\mathrm{delayed}\;\text{inflow}}}{+\;K_{1}S_{r}(t-\tau )I_{m}(t-\tau )+\;K_{3}V_{r}(t-\tau )I_{m}(t-\tau )}-(d_{r}+\gamma +\delta )I_{r}(t)+\rho V_{r}(t)+\gamma I_{r}(t)-d_{r}R_{r}(t). \end{array}$$

The current-incidence terms cancel with the delayed inflow terms when forming $N_{r}^{\prime }$ (as usual in infection models with or without delay); all vaccination and recovery transfers also cancel. These yields


$$N_{r}^{\prime }=\Lambda_{r}-d_{r}\;(S_{r}+V_{r}+I_{r}+R_{r})-\delta \;I_{r}=\Lambda_{r}-d_{r}N_{r}-\delta I_{r}\;\;\leq \;\;\Lambda_{r}-d_{r}N_{r}.$$

By comparison with $y^{\prime }=\Lambda_{r}-d_{r}y$, we obtain


$$N_{r}(t)\leq N_{r}(\text{0})e^{-d_{r}t}+\frac{\Lambda_{r}}{d_{r}}(\text{1}-e^{-d_{r}t})=\frac{\Lambda_{r}}{d_{r}}+(N_{r}(\text{0})-\frac{\Lambda_{r}}{d_{r}})e^{-d_{r}t}.$$

Similarly, summing the vector equations (6–7):


$$N_{m}^{\prime }=S_{m}^{\prime }+I_{m}^{\prime }=\Lambda_{m}-\lambda_{m}(t)S_{m}(t)-d_{m}S_{m}(t)+K_{\text{6}}S_{m}(t-\tau )I_{r}(t-\tau )-d_{m}I_{m}(t)=\Lambda_{m}-d_{m}\left(S_{m}+I_{m}\right)=\Lambda_{m}-d_{m}N_{m}.$$

so


$$N_{m}(t)=N_{m}(\text{0})e^{-d_{m}t}+\frac{\Lambda_{m}}{d_{m}}(\text{1}-e^{-d_{m}t})=\frac{\Lambda_{m}}{d_{m}}+(N_{m}(\text{0})-\frac{\Lambda_{m}}{d_{m}})e^{-d_{m}t}.$$

Both bounds imply $N_{r}(t)\leq \frac{\Lambda_{r}}{d_{r}}$ and $N_{m}(t)\leq \frac{\Lambda_{m}}{d_{m}}$ for all t≥0.

## 3 Equilibria of model and reproduction number $\left({ℛ}_{0}\right)$

In this section, we discussed the two different equilibria of model, Rift Valley Fever Free equilibria (RVFFE) and Rift Valley Fever endemic equilibrium (RVFEE) respectively.

### 3.1 Rift Valley fever–free equilibrium (RVFFE)

At the Rift Valley fever-free equilibrium, there is no infection in either population; hence $I_{r}^{\text{0}}=I_{m}^{\text{0}}=\text{0}$ and $R_{r}^{\text{0}}=\text{0}$. The equilibrium values for the other compartments are obtained by setting all time derivatives in (2–7) to zero and substituting $I_{r}=I_{m}=\text{0}$:


$$V_{\text{0}}=(S_{r}^{\text{0}},V_{r}^{\text{0}},I_{r}^{\text{0}},R_{r}^{\text{0}},S_{m}^{\text{0}},I_{m}^{\text{0}})=\left(\frac{\Lambda_{r}}{K_{\text{2}}},\;\;\frac{v\Lambda_{r}}{K_{\text{2}}K_{\text{4}}},\;\;\text{0},\;\;\text{0},\;\;\frac{\Lambda_{m}}{d_{m}},\;\;\text{0}\right),$$

where $K_{\text{2}}=v+d_{r}$ and $K_{\text{4}}=\rho +d_{r}$.

### 3.2 Rift Valley fever endemic equilibrium (RVFEE)

For the Rift Valley fever endemic equilibrium $V^{*}=(S_{r}^{*},V_{r}^{*},I_{r}^{*},R_{r}^{*},S_{m}^{*},I_{m}^{*})$, all compartments are positive ($I_{r}^{*},I_{m}^{*}>\text{0}$). Setting the derivatives in (2–7) to zero yields the following steady-state relations:


$$\begin{array}{cc} S_{r}^{*} & \;=\frac{\Lambda_{r}}{K_{\text{1}}I_{m}^{*}+K_{\text{2}}},\\ V_{r}^{*} & \;=\frac{vS_{r}^{*}}{(\text{1}-\alpha )K_{\text{1}}I_{m}^{*}+K_{\text{4}}},\\ I_{r}^{*} & \;=\frac{K_{\text{1}}S_{r}^{*}I_{m}^{*}+K_{\text{3}}V_{r}^{*}I_{m}^{*}}{K_{\text{5}}},\\ R_{r}^{*} & \;=\frac{\rho V_{r}^{*}+\gamma I_{r}^{*}}{d_{r}},\\ S_{m}^{*} & \;=\frac{\Lambda_{m}}{K_{\text{6}}I_{r}^{*}+d_{m}},\\ I_{m}^{*} & \;=\frac{K_{\text{6}}S_{m}^{*}I_{r}^{*}}{d_{m}}. \end{array}$$

Substituting the last relation into the fifth equation leads to a quadratic equation for $I_{m}^{*}$:


$$A_{\text{2}}(I_{m}^{*}{)}^{\text{2}}+A_{\text{1}}I_{m}^{*}+A_{\text{0}}=\text{0},$$

where,


$$\begin{array}{cc} A_{\text{2}} & \;=d_{m}K_{\text{1}}K_{\text{3}}(K_{\text{5}}+K_{\text{6}}\Lambda_{r}),\\ A_{\text{1}} & \;=d_{m}K_{\text{5}}(K_{\text{1}}K_{\text{4}}+K_{\text{2}}K_{\text{3}})+K_{\text{6}}\Lambda_{r}d_{m}(K_{\text{1}}K_{\text{4}}+K_{\text{3}}v-K_{\text{1}}K_{\text{3}}),\\ A_{\text{0}} & \;=d_{m}K_{\text{2}}K_{\text{4}}K_{\text{5}}-K_{\text{3}}K_{\text{6}}v\Lambda_{r}\Lambda_{m}. \end{array}$$

The positive root of this quadratic gives the biologically feasible endemic infection level $I_{m}^{*}>\text{0}$.

### 3.3 Basic reproduction number ($R_{0}$)

To compute the basic reproduction number $R_{\text{0}}$, we apply the next-generation matrix method, considering only the infectious classes of hosts ($I_{r}$) and mosquitoes ($I_{m}$).

From the linearized infected subsystem at the RVFFE:


$$[\begin{array}{c} I_{r}^{\prime }\\ I_{m}^{\prime } \end{array}]=[\begin{array}{cc} \text{0} & K_{\text{1}}S_{r}+K_{\text{3}}V_{r}\\ K_{\text{6}}S_{m} & \text{0} \end{array}][\begin{array}{c} I_{r}\\ I_{m} \end{array}]-[\begin{array}{cc} K_{\text{5}} & \text{0}\\ \text{0} & d_{m} \end{array}][\begin{array}{c} I_{r}\\ I_{m} \end{array}].$$

Hence, the next-generation matrix is


$$FV^{-\text{1}}=[\begin{array}{cc} \text{0} & \frac{K_{\text{1}}S_{r}+K_{\text{3}}V_{r}}{d_{m}}\\ \frac{K_{\text{6}}S_{m}}{K_{\text{5}}} & \text{0} \end{array}].$$

The spectral radius (dominant eigenvalue) of $FV^{-\text{1}}$ gives $R_{\text{0}}$:


$$\lambda^{\text{2}}=\frac{K_{\text{6}}S_{m}(K_{\text{1}}S_{r}+K_{\text{3}}V_{r})}{K_{\text{5}}d_{m}},\to R_{\text{0}}=\sqrt{\frac{K_{\text{6}}S_{m}(K_{\text{1}}S_{r}+K_{\text{3}}V_{r})}{K_{\text{5}}d_{m}}}.$$

Evaluating at the DFE $V_{\text{0}}$:


$$S_{r}=S_{r}^{\text{0}}=\frac{\Lambda_{r}}{K_{\text{2}}},V_{r}=V_{r}^{\text{0}}=\frac{v\Lambda_{r}}{K_{\text{2}}K_{\text{4}}},S_{m}=S_{m}^{\text{0}}=\frac{\Lambda_{m}}{d_{m}},$$

we obtain


$$R_{\text{0}}=\sqrt{\frac{K_{\text{6}}S_{m}^{\text{0}}(K_{\text{1}}S_{r}^{\text{0}}+K_{\text{3}}V_{r}^{\text{0}})}{K_{\text{5}}d_{m}}}$$

Substituting the parameter definitions


$$\begin{array}{cc} K_{\text{1}} & \;=b\beta_{r}e^{-d_{r}\tau },K_{\text{3}}=(\text{1}-\alpha )b\beta_{r}e^{-d_{r}\tau },\\ K_{\text{6}} & \;=b\beta_{m}e^{-d_{m}\tau },K_{\text{5}}=d_{r}+\gamma +\delta ,\\ K_{\text{2}} & \;=v+d_{r},K_{\text{4}}=\rho +d_{r}, \end{array}$$

gives the explicit expression


$$R_{\text{0}}=e^{-\frac{\text{1}}{\text{2}}(d_{r}+d_{m})\tau }\;b\;\sqrt{\frac{\beta_{r}\beta_{m}\;\Lambda_{r}\Lambda_{m}\;[(\text{1}-\alpha )v+K_{\text{4}}]}{(v+d_{r})(\rho +d_{r})(d_{r}+\gamma +\delta )\;d_{m}}}.$$

Thus, $R_{\text{0}}$ increases with the transmission rates $\beta_{r},\beta_{m}$ biting rate b, and recruitment rates $\Lambda_{r},\Lambda_{m}$; it decreases with natural mortality, recovery, and vaccination rates. The exponential factor $e^{-\frac{\text{1}}{\text{2}}(d_{r}+d_{m})\tau }$ reflects the damping effect of the delay on the infection potential.

## 4 Stability analysis

We study local stability of the delayed system (2–7) at the Rift Valley Fever–free equilibrium (RVFFE) and at the endemic equilibrium (RVFEE). Linearization of a retarded delay system $x^{\prime }(t)=A_{\text{0}}x(t)+A_{\text{1}}x(t-\tau )$ yields the characteristic equation


$$\text{det}(\lambda I-A_{\text{0}}-A_{\text{1}}e^{-\lambda \tau })=\text{0},$$

which is not a polynomial for τ>0.

The DFE is


$$V_{\text{0}}=(S_{r}^{\text{0}},V_{r}^{\text{0}},I_{r}^{\text{0}},R_{r}^{\text{0}},S_{m}^{\text{0}},I_{m}^{\text{0}})=(\frac{\Lambda_{r}}{K_{\text{2}}},\;\;\frac{v\Lambda_{r}}{K_{\text{2}}K_{\text{4}}},\;\;\text{0},\;\;\text{0},\;\;\frac{\Lambda_{m}}{d_{m}},\;\;\text{0}),$$

with $K_{\text{2}}=v+d_{r}$, $K_{\text{4}}=\rho +d_{r}$.

Define the forces of infection $\lambda_{r}(t)=b\beta_{r}I_{m}(t)$, $\lambda_{m}(t)=b\beta_{m}I_{r}(t)$ and the survival-adjusted constants


$$K_{\text{1}}=b\beta_{r}e^{-d_{r}\tau },K_{\text{3}}=(\text{1}-\alpha )b\beta_{r}e^{-d_{r}\tau },K_{\text{6}}=b\beta_{m}e^{-d_{m}\tau }.$$

Linearizing the infected subsystem $\left(I_{r},I_{m}\right)$ about $V_{\text{0}}$ gives


$$\begin{array}{cc} I_{r}^{\prime }(t) & \;=-K_{\text{5}}\;I_{r}(t)+a\;I_{m}(t-\tau ),\\ I_{m}^{\prime }(t) & \;=-d_{m}\;I_{m}(t)+b\;I_{r}(t-\tau ),a:=K_{\text{1}}S_{r}^{\text{0}}+K_{\text{3}}V_{r}^{\text{0}},\;\;\;\;b:=K_{\text{6}}S_{m}^{\text{0}}, \end{array}$$

with $K_{\text{5}}=d_{r}+\gamma +\delta$. The other coordinates are decoupled and stable (they only contain negative diagonal terms at the DFE).

The associated characteristic equation for the $\left(I_{r},I_{m}\right)$ block is


$$(\lambda +K_{\text{5}})(\lambda +d_{m})-ab\;e^{-\text{2}\lambda \tau }=\text{0}.$$

The next-generation approach (or the τ=0) gives


$$R_{\text{0}}=\sqrt{\frac{ab}{K_{\text{5}}d_{m}}}=\sqrt{\frac{(K_{\text{1}}S_{r}^{\text{0}}+K_{\text{3}}V_{r}^{\text{0}})\;K_{\text{6}}S_{m}^{\text{0}}}{K_{\text{5}}d_{m}}}.$$

***Theorem 3*** If $R_{\text{0}}<\text{1}$, the Rift Valley Fever free equilibrium $V_{\text{0}}$ is locally asymptotically stable for all τ≥0. If $R_{\text{0}}>\text{1}$, $V_{\text{0}}$ is unstable for all τ≥0.

***Proof:*** Consider the linear DDE $x^{\prime }(t)=-Dx(t)+Bx(t-\tau )$ with $D=\text{diag}(K_{\text{5}},d_{m})$ and $B=[\begin{array}{cc} \text{0} & a\\ b & \text{0} \end{array}]$. Its characteristic equation is (9). A standard delay-independent stability result for retarded systems states that if the spectral radius $\rho (D^{-\text{1}}B)<\text{1}$, then all characteristic roots satisfy ℜλ<0 for every τ≥0; conversely, if $\rho (D^{-\text{1}}B)>\text{1}$, instability holds for every τ≥0. Here,


$$\rho (D^{-\text{1}}B)=\sqrt{\frac{ab}{K_{\text{5}}d_{m}}}=R_{\text{0}},$$

hence the claim. (Equivalently, when τ=0 the roots solve $(\lambda +K_{\text{5}})(\lambda +d_{m})-ab=\text{0}$; stability holds iff $ab<K_{\text{5}}d_{m}$, i.e., $R_{\text{0}}<\text{1}$. The delay perturbs the roots continuously without crossing the imaginary axis under $R_{\text{0}}<\text{1}$.)

***Theorem 4*** If $\begin{array}{c} R_{\text{0}}>\text{1} \end{array}$ then the system is stable at Rift Vally Fever endemic equilibrium $\left(RVFEE-V^{*}\right)=V^{*}=\left(S_{r}^{*},\;V_{r}^{*},\;I_{r}^{*},R_{r}^{*},S_{m}^{*},I_{m}^{*}\right)$ in the sense of local. Otherwise, if $\begin{array}{c} R_{\text{0}}>\text{1} \end{array}$ then the system loses its stability.

***Proof:*** Let $V^{*}=(S_{r}^{*},V_{r}^{*},I_{r}^{*},R_{r}^{*},S_{m}^{*},I_{m}^{*})$ denote an endemic equilibrium (when it exists, typically for $R_{\text{0}}>\text{1}$). Linearization yields a system of the form


$$x^{\prime }(t)=A_{\text{0}}^{*}x(t)+A_{\text{1}}^{*}x(t-\tau ),$$

so, the characteristic equation is


$$\text{det}(\lambda I-A_{\text{0}}^{*}-A_{\text{1}}^{*}e^{-\lambda \tau })=\text{0},$$ (8)

again, transcendental for τ>0. Unlike the RVFFE, delay can now induce stability switches (Hopf bifurcations) as τ varies, because infection-to-infection feedback is present at $V^{*}$.

- Benchmark (τ=0). Equation (8) reduces to a polynomial; Routh–Hurwitz criteria can be applied to assess local stability at $V^{*}$ (this is the only setting where the polynomial approach is valid).
- Delay (τ>0). A Hopf crossing λ=iω(ω>0) satisfies

$$\text{det}(\text{i}\omega I-A_{\text{0}}^{*}-A_{\text{1}}^{*}e^{-\text{i}\omega \tau })=\text{0}.$$

For the infected 2 × 2 block this reduces to


$$(\text{i}\omega +\kappa_{\text{1}})(\text{i}\omega +\kappa_{\text{2}})-a_{*}b_{*}e^{-\text{2}\text{i}\omega \tau }=\text{0},$$

with $\kappa_{\text{1}},\kappa_{\text{2}}>\text{0}$ and $a_{*},b_{*}>\text{0}$ evaluated at $V^{*}$. Taking moduli gives the frequency condition


$$(\omega^{\text{2}}+\kappa_{\text{1}}^{\text{2}})(\omega^{\text{2}}+\kappa_{\text{2}}^{\text{2}})=(a_{*}b_{*}{)}^{\text{2}},$$

and the phase condition yields the corresponding critical delays


$$\tau_{k}^{*}=\frac{\text{1}}{\text{2}\omega }(\text{arg}((\text{i}\omega +\kappa_{\text{1}})(\text{i}\omega +\kappa_{\text{2}}))+\text{2}\pi k),k\in Z_{\geq \text{0}}.$$

Thus, for sufficiently small τ the RVFEE inherits the τ=0 stability; increasing τ can generate oscillations via Hopf bifurcation.

***Theorem 5.*** Assume nonnegative, bounded history on [−τ,0] for all state variables. If $R_{\text{0}}<\text{1}$, then the Rift Valley Fever free equilibrium


$$V_{\text{0}}=(\frac{\Lambda_{r}}{K_{\text{2}}},\frac{v\Lambda_{r}}{K_{\text{2}}K_{\text{4}}},\;\text{0},\;\text{0},\;\frac{\Lambda_{m}}{d_{m}},\;\text{0})$$

is globally asymptotically stable.

***Proof:*** Define Lyapunov–Krasovskii functional. Use the standard convex “entropy” terms for the noninfected classes and add delay integrals that exactly cancel the delayed inflows into $I_{r}$ and $I_{m}$:


$$\Phi (x;x^{\text{0}}):=x-x^{\text{0}}-x^{\text{0}}\text{ln}(\frac{x}{x^{\text{0}}})\geq \text{0},$$

$$\begin{array}{cc} U(t):=\; & \;\Phi \left(S_{r};S_{r}^{\text{0}}\right)+\Phi \left(V_{r};V_{r}^{\text{0}}\right)+\Phi \left(S_{m};S_{m}^{\text{0}}\right)\;+\;I_{r}(t)+I_{m}(t)+R_{r}(t)+\frac{\text{1}}{K_{\text{5}}}\int_{t-\tau }^{t}\left(K_{\text{1}}S_{r}(u)I_{m}(u)+K_{\text{3}}V_{r}(u)I_{m}(u)\right)\;du\\ & \;+\frac{\text{1}}{d_{m}}\int_{t-\tau }^{t}K_{\text{6}}S_{m}(u)I_{r}(u)\;du. \end{array}$$

Using the model equations, the identities


$$\frac{d}{dt}\Phi (S_{r};S_{r}^{\text{0}})=(\text{1}-\frac{S_{r}^{\text{0}}}{S_{r}}){\dot{S}}_{r},\;\;\frac{d}{dt}\int_{t-\tau }^{t}g(u)\;du=g(t)-g(t-\tau ),$$

and the RVFFE equalities $\Lambda_{r}=K_{\text{2}}S_{r}^{\text{0}}$, $\Lambda_{m}=d_{m}S_{m}^{\text{0}}$, one obtains after cancellations:


$$\begin{array}{cc} & \;\dot{U}\leq -\frac{\Lambda_{r}}{S_{r}^{\text{0}}}\;\frac{(S_{r}-S_{r}^{\text{0}}{)}^{\text{2}}}{S_{r}}-\frac{vS_{r}}{V_{r}^{\text{0}}}\;\frac{(V_{r}-V_{r}^{\text{0}}{)}^{\text{2}}}{V_{r}}-\frac{\Lambda_{m}}{S_{m}^{\text{0}}}\;\frac{(S_{m}-S_{m}^{\text{0}}{)}^{\text{2}}}{S_{m}}-\delta \;I_{r}-d_{r}R_{r}-\left(K_{\text{5}}-\frac{\left(K_{\text{1}}S_{r}^{\text{0}}+K_{\text{3}}V_{r}^{\text{0}}\right)K_{\text{6}}S_{m}^{\text{0}}}{d_{m}}\right)I_{r}\\ & \;-\left(d_{m}-\frac{\left(K_{\text{1}}S_{r}^{\text{0}}+K_{\text{3}}V_{r}^{\text{0}}\right)K_{\text{6}}S_{m}^{\text{0}}}{K_{\text{5}}}\right)I_{m}. \end{array}$$

Because


$$\frac{(K_{\text{1}}S_{r}^{\text{0}}+K_{\text{3}}V_{r}^{\text{0}})K_{\text{6}}S_{m}^{\text{0}}}{K_{\text{5}}d_{m}}=R_{\text{0}}^{\text{2}}<\text{1},$$

the coefficients of $I_{r}$ and $I_{m}$ above are strictly positive, hence


$$\dot{U}\leq \;-c_{\text{1}}\frac{(S_{r}-S_{r}^{\text{0}}{)}^{\text{2}}}{S_{r}}-c_{\text{2}}\frac{(V_{r}-V_{r}^{\text{0}}{)}^{\text{2}}}{V_{r}}-c_{\text{3}}\frac{(S_{m}-S_{m}^{\text{0}}{)}^{\text{2}}}{S_{m}}-c_{\text{4}}I_{r}-c_{\text{5}}I_{m}-\delta I_{r}-d_{r}R_{r}.$$

for some constants $c_{i}>\text{0}$. Therefore $\dot{U}\leq \text{0}$, and $\dot{U}=\text{0}$ only at


$$S_{r}=S_{r}^{\text{0}},\;\;V_{r}=V_{r}^{\text{0}},\;\;S_{m}=S_{m}^{\text{0}},\;\;I_{r}=I_{m}=R_{r}=\text{0},$$

i.e., at the Rift Valley Fever free equilibrium. By LaSalle’s invariance principle for retarded functional differential equations, trajectories converge to $V_{\text{0}}$.

***Theorem 6*.** *Assume* τ=0*. If* $R_{\text{0}}>\text{1}$*, then the Rift Valley Fever endemic equilibrium*


$$V^{*}=(S_{r}^{*},V_{r}^{*},I_{r}^{*},R_{r}^{*},S_{m}^{*},I_{m}^{*})$$

*exists and is globally asymptotically stable in the interior of the feasible region* V*.*

*Proof. When* τ=0*, system (2–7) becomes in the form:*


$$\dot{X}=F(X),X=(S_{r},V_{r},I_{r},R_{r},S_{m},I_{m})\in R_{+}^{\text{6}}.$$

*Define the standard Volterra (entropy) function*


$$\Phi (x;x^{*})\;\;=\;\;x-x^{*}-x^{*}ln(\frac{x}{x^{*}})\geq \text{0}\;\text{for}\;x>\text{0}).$$

*Consider the Lyapunov function*

$L(t)=\Phi (S_{r};S_{r}^{*})+\Phi (V_{r};V_{r}^{*})+\Phi (I_{r};I_{r}^{*})+\Phi (R_{r};R_{r}^{*})+\Phi (S_{m};S_{m}^{*})+\Phi (I_{m};I_{m}^{*}).$ *Then* L(t)≥0*, and* L(t)=0 *iff* $X(t)=V^{*}$.

$\dot{L}(t)=(\text{1}-\frac{S_{r}^{*}}{S_{r}}){\dot{S}}_{r}+(\text{1}-\frac{V_{r}^{*}}{V_{r}}){\dot{V}}_{r}+(\text{1}-\frac{I_{r}^{*}}{I_{r}}){\dot{I}}_{r}+(\text{1}-\frac{R_{r}^{*}}{R_{r}}){\dot{R}}_{r}+(\text{1}-\frac{S_{m}^{*}}{S_{m}}){\dot{S}}_{m}+(\text{1}-\frac{I_{m}^{*}}{I_{m}}){\dot{I}}_{m}.$

*At the endemic equilibrium* $V^{*}$, by setting ${\dot{S}}_{r}={\dot{V}}_{r}={\dot{I}}_{r}={\dot{R}}_{r}={\dot{S}}_{m}={\dot{I}}_{m}=\text{0}$*, the following identities hold (these are just the steady-state balances):*

- *Infection–removal balance in ruminants:*

$$(K_{\text{1}}S_{r}^{*}+K_{\text{3}}V_{r}^{*})\;I_{m}^{*}=K_{\text{5}}\;I_{r}^{*}.$$

- *Infection–removal balance in mosquitoes:*

$$K_{\text{6}}S_{m}^{*}\;I_{r}^{*}=d_{m}\;I_{m}^{*}.$$

*After substitution and grouping terms,* $\dot{L}$ *can be written as a sum of expressions of the form*


$$c\;(\text{2}-\frac{x}{x^{*}}-\frac{x^{*}}{x}),$$

*and mixed infection terms that can be reorganized into products like*


$$c\;(\text{1}-\frac{S_{r}^{*}}{S_{r}})(\text{1}-\frac{I_{m}^{*}}{I_{m}}),$$

*which are controlled using the inequality*


u−1−lnu≥0 for u>0,

*equivalently,*


$$\text{1}-\frac{\text{1}}{u}\leq ln\;u.$$

*Concretely, the infection part can be bounded by applying the arithmetic–geometric mean inequality in the standard epidemic Lyapunov style. One obtains:*


$$\dot{L}(t)\leq -C_{\text{1}}\;\frac{(S_{r}-S_{r}^{*}{)}^{\text{2}}\;}{S_{r}}-C_{\text{2}}\;\frac{(V_{r}-V_{r}^{*}{)}^{\text{2}}\;}{V_{r}}-C_{\text{3}}\;\frac{(I_{r}-I_{r}^{*}{)}^{\text{2}}\;}{I_{r}}-C_{\text{4}}\;\frac{(R_{r}-R_{r}^{*}{)}^{\text{2}}\;}{R_{r}}-C_{\text{5}}\;\frac{(S_{m}-S_{m}^{*}{)}^{\text{2}}\;}{S_{m}}-C_{\text{6}}\;\frac{(I_{m}-I_{m}^{*}{)}^{\text{2}}\;}{I_{m}},$$

*for some constants* $C_{i}>\text{0}\;$*(these constants are functions of parameters and equilibrium values and are strictly positive because* $V^{*}\in int\left(\text{V}\right)$*).*

*Therefore,*


$$\dot{L}(t)\leq \text{0}\;for\;all\;t\geq \text{0}.$$

*If* $\dot{L}(t)=\text{0}$*, then each nonpositive term above must be zero, implying*


$$S_{r}=S_{r}^{*},\;\;V_{r}=V_{r}^{*},\;\;I_{r}=I_{r}^{*},\;\;R_{r}=R_{r}^{*},\;\;S_{m}=S_{m}^{*},\;\;I_{m}=I_{m}^{*}.$$

*Hence the only invariant set contained in* $\left\{\dot{L}=\text{0}\right\}$ *is the singleton* $\left\{V^{*}\right\}$*.*

*Since solutions are bounded and remain in* V*, and* L(t) *is nonincreasing and radially well-behaved on* V*, by LaSalle’s invariance principle, every trajectory with positive initial data satisfies*


$$\underset{t\to \infty }{lim}X(t)=V^{*}.$$

*Thus* $V^{*}$ *is globally asymptotically stable in* int(V)*.*

## 5 Sensitivity analysis

**Fig 2:**
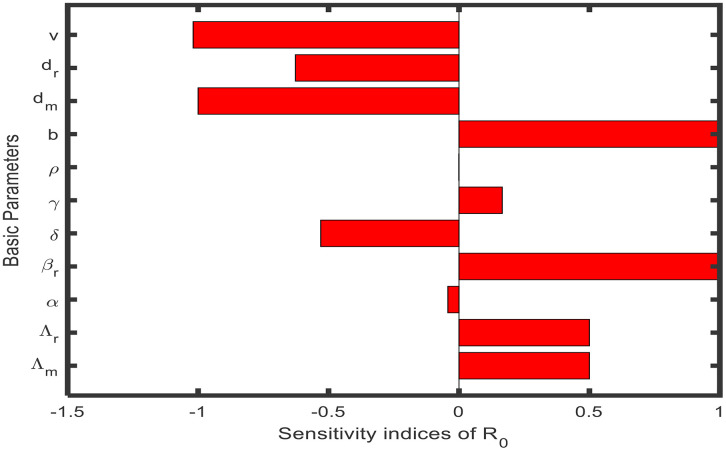
Sensitivity indices of reproduction number $\left({𝐑}_{\text{0}}\right)$.

In this section, the behavior of model parameters concerning reproduction number $\begin{array}{c} R_{\text{0}} \end{array}$ will be examined (Fig 2). Also, we examine the transmission and spread of disease with the sensitive analysis of the model. Preliminary: The formalized sensitivity index of a variable e, that depends differentiable on a parameter ϱ:


$$\begin{array}{c} E_{\varrho }^{℮}=\frac{\varrho }{℮}\times \frac{\partial ℮}{\partial \varrho } \end{array}$$

In spatial terms, determine the sensitive indices of parameters concerning the reproduction number $\begin{array}{c} R_{\text{0}} \end{array}$.

$V_{b}=\frac{b}{R_{\text{0}}}\times \frac{\partial R_{\text{0}}}{\partial b}=\text{1}>\text{0}$, $V_{\beta_{r}}=\frac{\beta_{r}}{R_{\text{0}}}\times \frac{\partial R_{\text{0}}}{\partial \beta_{r}}=\text{1}>$,$V_{d_{m}}=\frac{d_{m}}{R_{\text{0}}}\times \frac{\partial R_{\text{0}}}{\partial d_{m}}=-\text{1}<\text{0}$, $V_{\Lambda_{m}}=\frac{\Lambda_{m}}{R_{\text{0}}}\times \frac{\partial R_{\text{0}}}{\partial \Lambda_{m}}=\frac{\text{1}}{\text{2}}>\text{0}$,$V_{\Lambda_{r}}=\frac{\Lambda_{r}}{R_{\text{0}}}\times \frac{\partial R_{\text{0}}}{\partial \Lambda_{r}}=\frac{\text{1}}{\text{2}}>\text{0}$, $V_{\alpha }=\frac{\alpha }{R_{\text{0}}}\times \frac{\partial R_{\text{0}}}{\partial \alpha }=\frac{-\alpha \left(\rho +d_{r}+\text{2}\gamma (\text{1}-\alpha )\right)}{\text{2}(\text{1}-\alpha )\left(\rho +d_{r}+\gamma (\text{1}-\alpha )\right)}<\text{0}$, $V_{\rho }=\frac{\rho }{R_{\text{0}}}\times \frac{\partial R_{\text{0}}}{\partial \rho }=\frac{-\gamma (\text{1}-\alpha )\rho }{\text{2}\left(\rho +d_{r})(\rho +d_{r}+\gamma (\text{1}-\alpha )\right)}<\text{0}$, $V_{\gamma }=\frac{\gamma }{R_{\text{0}}}\times \frac{\partial R_{\text{0}}}{\partial \gamma }=\frac{\gamma (\text{1}-\alpha )\left(\delta +d_{r}\right)}{\text{2}\left(\gamma +\delta +d_{r}\right)\left(\rho +d_{r}+\gamma (\text{1}-\alpha )\right)}>\text{0}$, $V_{v}=\frac{v}{R_{\text{0}}}\times \frac{\partial R_{\text{0}}}{\partial v}=\frac{-\text{1}}{v+d_{r}}<\text{0}$, $V_{\delta }=\frac{\delta }{R_{\text{0}}}\times \frac{\partial R_{\text{0}}}{\partial \delta }=\frac{-\text{1}}{\gamma +\delta +d_{r}}<\text{0}$,


$$V_{d_{r}}=\frac{d_{r}}{R_{\text{0}}}\times \frac{\partial R_{\text{0}}}{\partial d_{r}}=\frac{\text{1}}{\text{2}}\left(\frac{d_{r}}{A}-\frac{d_{r}}{K_{\text{5}}}-\text{1}-\frac{d_{r}}{B}-d_{r}\tau \right).$$

Where $A=\left(\rho +d_{r}\right)+(\text{1}-\alpha )v$, $B=v+\rho +d_{r},K_{\text{5}}=d_{r}+\gamma +\delta$

The uncertainty signs of the sensitivity indicators are presented in Table 2. In Table 2, the more sensitive parameters are shown with positive sign of the uncertainty indicators of $R_{\text{0}}$, whilst the less sensitive parameters are shown with a non-positive sign of the sensitivity indicators of the reproduction number $R_{\text{0}}$. Furthermore, the disease will be managed in the population if we control the highly sensitive transmission parameter $"\beta_{r}"$.

**Table 2:** Parameters sensitivity signs.

Parameters	Values	Signs
$\beta_{r}$	1	Positive
b	1	Positive
$d_{m}$	−1	Negative
$d_{r}$	−0.626942	Negative
$\Lambda_{m}$	0.5	Positive
$\Lambda_{r}$	0.5	Positive
α	−0.0427	Negative
ρ	−0.0006	Negative
γ	0.166	Positive
v	−1.019	Negative
δ	−0.53	Negative

## 6 Results and discussion

In this study, the delay differential equation (DDE) system (2–7) was numerically solved using the built-in MATLAB solver DDE23, which is based on an adaptive step-size Runge–Kutta method suitable for both stiff and non-stiff delay systems. The solver was employed to simulate system (2–7) and visualize the time-dependent behavior of each compartment using the parameter values obtained from the scientific literature, as listed in Table 1.

The simulations were performed over the time interval t∈[0,50] with a relative tolerance of ${\text{1}\text{0}}^{-\text{6}}$and an initial step size of Δt=0.01. These settings provided a stable and accurate numerical approximation of the model trajectories. The initial conditions were chosen based on biologically plausible estimates of Rift Valley Fever (RVF) dynamics: the majority of the ruminants were initially susceptible, a small percentage vaccinated, and a few infected, while the mosquito population began overwhelmingly susceptible. This setup is the beginning stage of an outbreak after introducing a finite number of infected vectors.

**Fig 3:**
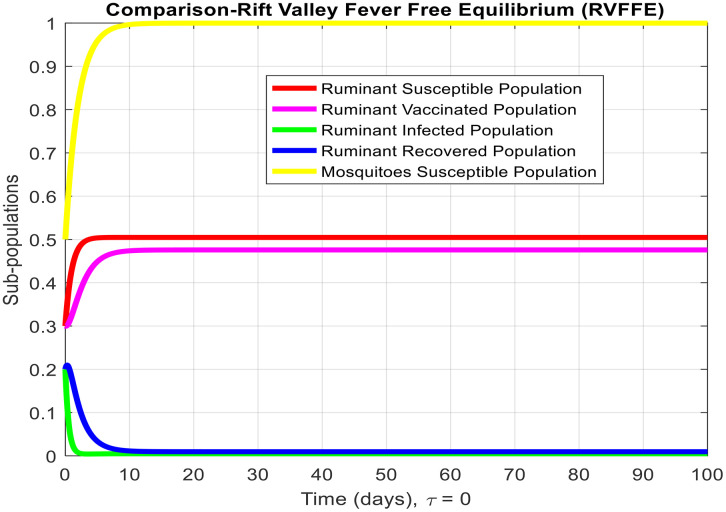
Subpopulation graph containing the time of the system (2-7) at the Rift Valley Fever-free equilibrium of the model whenτ=0. (This is deterministic case to understand the effect of delay in other results).

**Fig 4:**
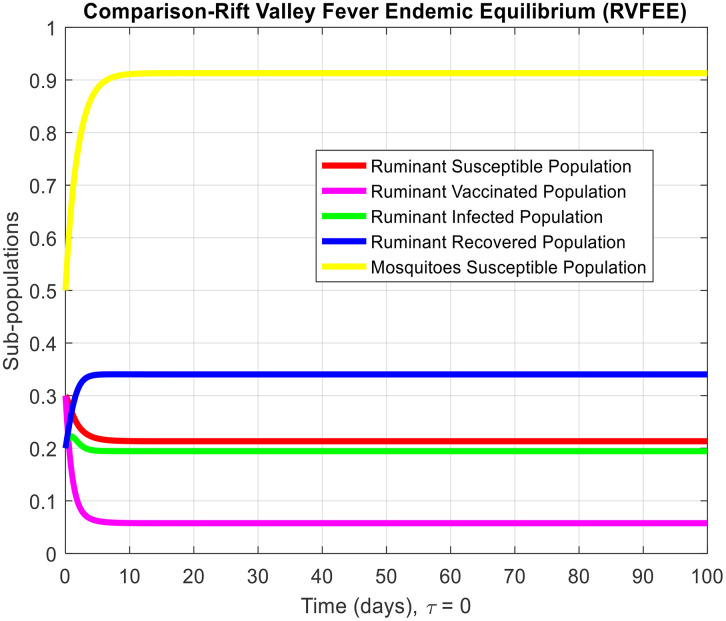
Subpopulation graph containing the time of the system (2-7) at the Rift Valley Fever- endemic equilibrium of the model when τ=0. (This is deterministic case to understand the effect of delay in other results).

**Fig 5:**
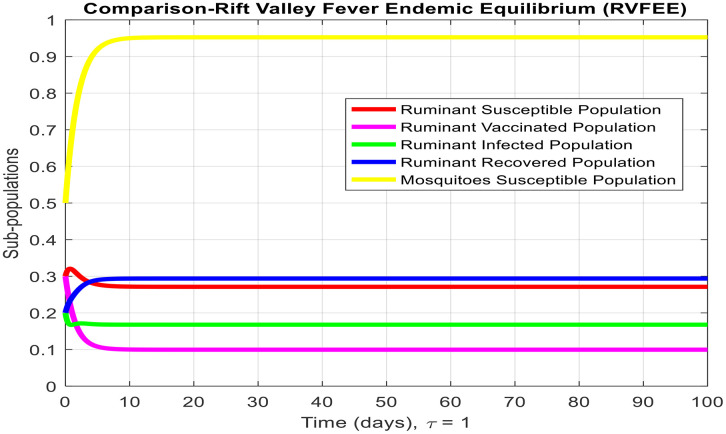
Subpopulation graph containing the time of the system at Rift Valley Fever endemic equilibrium with the value of delay tactics like at τ=1.

**Fig 6:**
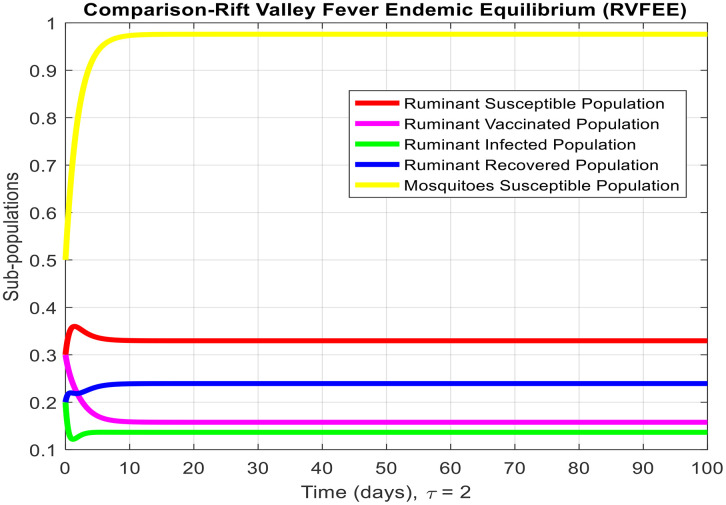
Subpopulation graph containing the time of the system at Rift Valley Fever endemic equilibrium with the value of delay tactics like at τ=2.

Figs 3-6 demonstrate temporal dynamics of the ruminant and mosquito subpopulations for different parameter values. Although the delay may reduce the effective transmission potential (through survival-adjusted terms) and facilitate the elimination of disease when $R_{\text{0}}<\text{1}$, it cannot guarantee the stability of all regimes. First, because this technology has just emerged $R_{\text{0}}>\text{1}$, increasing τ can support the creation of oscillations via the Hopf bifurcation at the equilibrium point of the endemic equilibrium, which is a known property of the delayed SIR model. Thus, the interpretation of the role of delay requires some caution: delay can act to damp the infection, but it can also cause more persistent cyclic outbreaks.

These findings coincide with analytical findings on equilibria stability and highlight the pivotal role of $R_{\text{0}}$ in disease persistence determination. From Fig 3 and 4, we observe the behavior of Rift Valley fever around the disease-free and endemic equilibria, respectively, when the time delay is set to zero, illustrating the deterministic behavior of the model.

**Fig 7:**
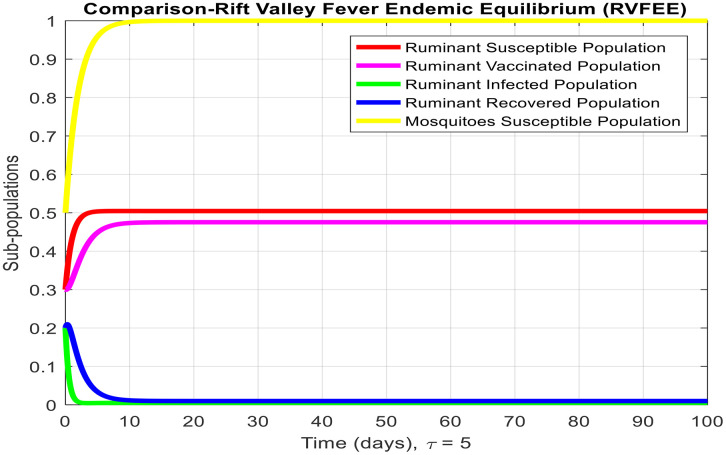
Subpopulation graph containing the time of the system at Rift Valley Fever endemic equilibrium with the value of delay tactics like at τ=5.

**Fig 8:**
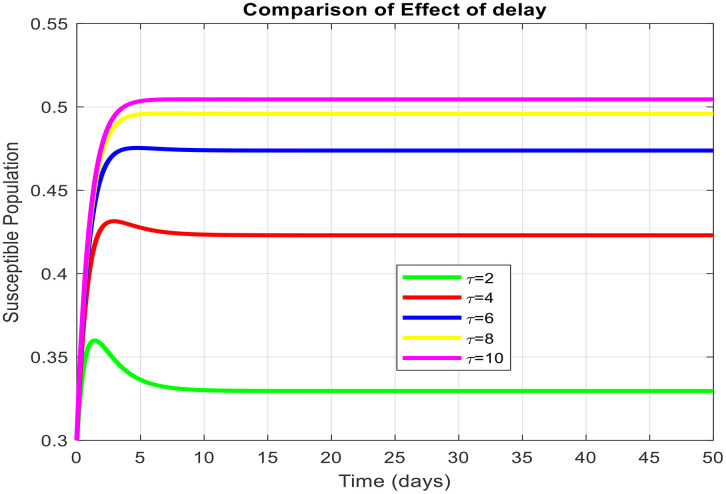
Effect of delay parameters at susceptible populations on the dynamics of virus with the different values of delay tactics.

**Fig 9:**
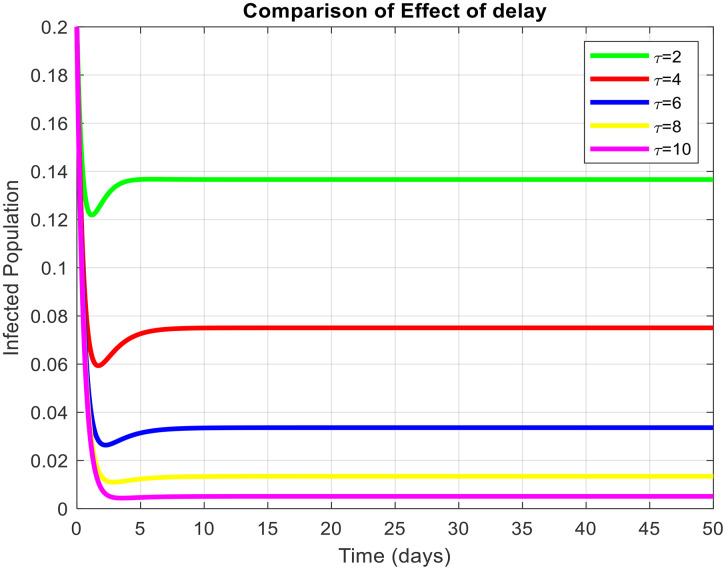
Effect of delay parameters at infected populations on the dynamics of virus with the different values of delay tactics.

τdecreases the effective contact among susceptible and infected populations and hence reduces the overall transmission capacity. This is due to the reason that the delay parameter plays an essential role in infection amplitude and convergence rate towards equilibrium. From Figs 5, 6 and 7, it is clear that for τ=1,2 and 5, the susceptible population grows and the infected population asymptotically approaches towards zero. This asymptotically approaches towards zero for large delays. Figs 8 and 9 also show the impact of delay values (τ=2,4,6,8,10) on the infected class and the susceptible class, respectively, proving how higher delays reduce the infection dynamics and make disease eradication easier.

Entropy-based sensitivity analysis, as shown in **Fig 2**, provides an estimate of the relative ranking of key parameters on the basic reproduction number $R_{\text{0}}$. Sensitivity values that are positive represent parameters that favor disease spread (e.g., biting rate band infection rates $\beta_{r}$, $\beta_{m}$), whereas negative sensitivity values represent parameters that suppress spread (e.g., recovery rate γ and natural mortality rates $d_{r}$, $d_{m}$). The analysis provides a quantitative importance ranking of the parameters and helps design targeted control measures.

**Fig 10:**
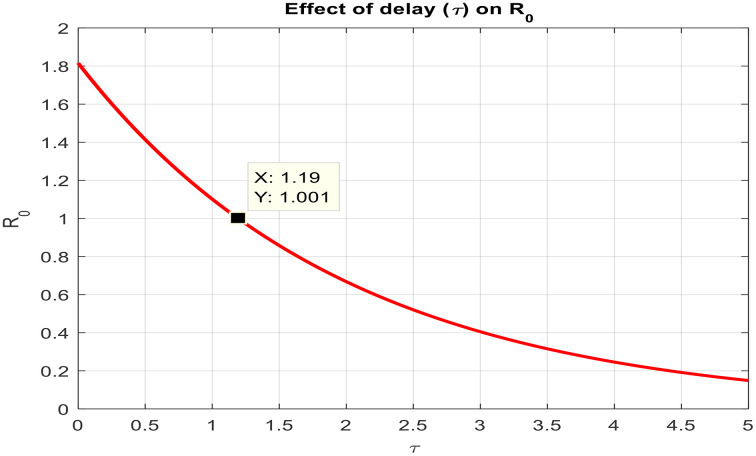
Effect of time delay (τ) on Reproduction number $\left({𝐑}_{\text{0}}\right)$.

Finally, Fig 10 shows how the delay parameter τ and the reproduction number $R_{\text{0}}\;$ interact. As τ increases, $R_{\text{0}}$ decreases, indicating that longer incubation or transmission delays lower the epidemic potential of RVF. Over time, the infection eventually vanishes from the population, confirming the theoretical prediction that sufficient delay can stabilize the system and lead to disease eradication.

Biologically, as the delay time increases, the basic reproduction ratio gets smaller by restricting the transmission rates during the incubation or progression periods, since the infected hosts and vectors, in this case, are not immediately contributing to the spread of the disease and can also die without becoming a potential transmitter of the disease. In the case of Rift Valley Fever, the delays can be the immune response and the effects of intervention, which can also reduce the host-vector transmission cycle and move the system from the state of endemics towards disease elimination.

Overall, these numerical experiments validate the analytical results and demonstrate that the entropy-based global stability framework effectively captures the influence of delay, parameter sensitivity, and control mechanisms on the dynamics of Rift Valley Fever transmission.

The strategy indicated to be most effective in controlling RVF would be to integrate vaccination of ruminants with measures to reduce the bite rate of mosquitoes. This would result in a reduction in the density of the vectors. Additionally, vaccination of livestock will result in a reduction in the number of animals $R_{\text{0}}$, slow down the transmission, and can change the disease system from Endemic Persistence to Disease Elimination.

## 7 Conclusion and future remarks

In this study, a delayed nonlinear mathematical model for Rift Valley Fever (RVF) transmission was analyzed to investigate the effects of time delay and parameter sensitivity on disease dynamics. The ruminant population was divided into four compartments: susceptible ($S_{r}$), vaccinated ($V_{r}$), infected ($I_{r}$), and recovered ($R_{r}$), while the female mosquito population was divided into susceptible ($S_{m}$) and infected ($I_{m}$) classes. These six interacting subpopulations form the nucleus of the proposed model.

The dynamical analysis addressed fundamental properties of the model, including positivity, boundedness, existence of equilibria, and threshold conditions. The basic reproduction number ($R_{\text{0}}$) was estimated through the next-generation matrix method, and sensitivity to critical parameters was examined through an entropy-based sensitivity analysis that provided a quantitative measure of parameter impact on disease persistence.

Global stability of equilibria was examined through Lyapunov and LaSalle invariance principles in order to identify conditions under which the system converges to the disease-free or endemic equilibrium. The global stability theory based on entropy also illustrated how delay and uncertainty act and affect long-term dynamics of the epidemic system. Numerical simulations, carried out using MATLAB’s internal DDE23 solver, verified the theoretical results and demonstrated dynamic effects of delay, vaccination, and parameter fluctuation on the transmission process.

From an epidemiological perspective, the findings emphasize the augmentation of the time delay (τ) and improvement in control interventions such as vaccination and recovery in reducing $R_{\text{0}}\;$and causing disease elimination efficiently. This indicates the effectiveness of entropy-based global stability analysis as a useful tool in comprehending and controlling vector-borne disease.

Additional studies would complement this work by incorporating spatial diffusion, environmental heterogeneity, and randomness to simulate more realistic transmission scenarios. Integrating actual epidemiologic data into the model and studying optimal control policies using entropy-based optimization schemes would also be beneficial to prevent and control Rift Valley Fever and other emerging infectious diseases.
